# Mid-IR evanescent-field fiber sensor with enhanced sensitivity for volatile organic compounds†

**DOI:** 10.1039/c9ra04104d

**Published:** 2019-07-08

**Authors:** Farah Alimagham, Max Platkov, Joshua Prestage, Svetlana Basov, Gregory Izakson, Abraham Katzir, Stephen R. Elliott, Tanya Hutter

**Affiliations:** Department of Chemistry, University of Cambridge Cambridge CB2 1EW UK tf269@cam.ac.uk; Nuclear Research Center Negev Beer-Sheva 84190 Israel; SensorHut Ltd Cambridge CB2 9DN UK; Department of Biomedical Engineering, The Aby and Aladar Fleischman Faculty of Engineering, Tel-Aviv University Tel-Aviv 69987 Israel; Raymond and Beverly Sackler Faculty of Exact Science, School of Physics and Astronomy, Tel-Aviv University Tel-Aviv 69987 Israel

## Abstract

The increasing awareness of the harsh environmental and health risks associated with air pollution has placed volatile organic compounds (VOCs) sensor technologies in elevated demand. While the currently available VOC-monitoring technologies are either bulky and expensive, or only capable of measuring a total VOC concentration, the selective detection of VOCs in the gas-phase remains a challenge. To overcome this, a novel method and device based on mid-IR evanescent-wave fiber-optic spectroscopy, which enables enhanced detection of VOCs, is hereby proposed. This is achieved by increasing the number of analyte molecules in the proximity of the evanescent field *via* capillary condensation inside nano-porous microparticles coated on the fiber surface. The nano-porous structure of the coating allows the VOC analytes to rapidly diffuse into the pores and become concentrated at the surface of the fiber, thereby allowing the utilization of highly sensitive evanescent-wave spectroscopy. To ascertain the effectiveness and performance of the sensor, different VOCs are measured, and the enhanced sensitivity is analyzed using a custom-built gas cell. According to the results presented here, our VOC sensor shows a significantly increased sensitivity compared to that of an uncoated fiber.

## Abstract 2

Increased sensitivity of mid-IR evanescent field sensing for gas-phase volatile organic compound detection using a nano-porous coating of an optical-fibre.

## Graphical abstract

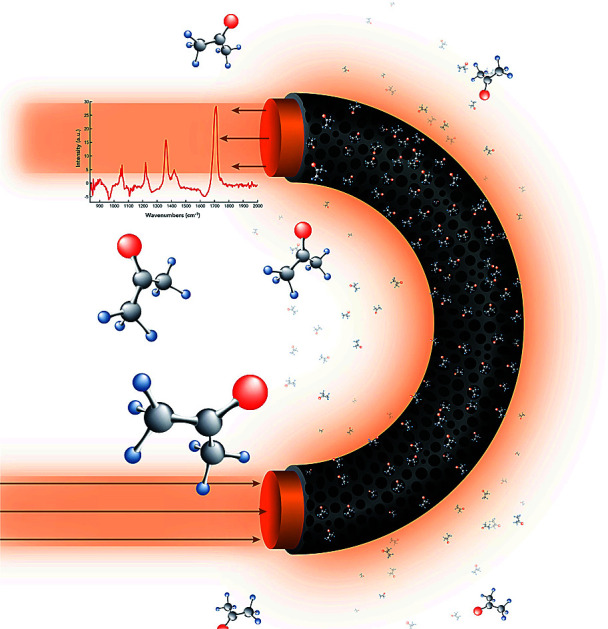

## Introduction

Volatile organic compounds (VOCs) are a group of carbon-based chemical compounds which are prone to evaporate under ambient conditions. The detection, monitoring and analysis of VOC gases is crucial for many applications, for example industrial and agricultural ones, security, and scientific research. There is a strong need for the ability to detect toxic gases, traces of explosives, as well as indoor and outdoor air quality. Studies have shown that the inhalation of excess VOCs and their degradation products is linked to respiratory-system damage and even cancer.^1^ In addition, human safety is potentially threatened by the toxicity, high flammability and explosivity of certain VOCs. Besides human health and safety, VOCs may also be related to major environmental problems, such as global warming, stratospheric ozone depletion and photochemical ozone formation.^2^ Therefore, the development of new technologies that can enable highly effective sensors to monitor VOC gases in various application fields is of great importance. Currently available low-cost sensors for VOCs are based on the use of metal oxide semiconductors and photo-ionization principles.^3^ These detectors are only capable of measuring the total VOC concentration, without any ability to differentiate between them. For any specific VOC detection and classification, bulky systems (such as gas-chromatography mass-spectrometry) and complex sample preparation are required,^4,5^ making them unsuitable for real-time, in-line and *in situ* VOC monitoring.

The mid-infrared (mid-IR) spectral range extends from 2.5 to 25 μm (4000 to 400 cm^−1^) and offers the unique possibility to provide quantitative compositional, chemical and structural information on a wide range of molecules due to the specific excitation of vibrational and rotational transitions of these compounds in the gas, liquid and solid phases. Therefore, spectroscopy in the mid-IR has emerged as an attractive alternative for molecular selectivity, fast acquisition times, real-time monitoring, high sensitivity and direct label-free analysis in relatively complex media, thus offering diverse applications, ranging from medical, industrial production monitoring, environmental analysis and material science. The development and miniaturization of advanced mid-IR light-source technologies, such as quantum cascade lasers (QCLs) and inter-band cascade lasers (ICLs), have enabled improved performance of evanescent-field based sensors, including attenuated total reflectance (ATR) crystals, optical fibers and waveguides.^6,7^ The evanescent field is the fraction of the guided light outside of the waveguide, which is generated at the interface between an optically denser waveguide and an optically thinner adjacent medium if the condition of total internal reflection (TIR) is fulfilled. The external medium and absorbing species present within the penetration depth of the evanescent field interact with the guided light, resulting in an attenuation at the frequencies where resonant energy transitions may occur. Since the penetration depth of the evanescent field depends on the wavelength, evanescent-field sensing based on broadband light sources results in a distribution of penetration depths, while for single-frequency lasers or narrow-band emission, the penetration depth is well characterized. In the mid-IR spectral range, the penetration depth of many common and relevant materials is of the order of a few microns. For example, for wavelengths of *λ* = 5 and 25 μm, for a silver halide waveguide with *n*_1_ = 2.1, *θ* = 50°, the penetration depth, *d*_p_, in air is between 1 and 5 μm, respectively. Fiber-optic and waveguide based evanescent-wave sensors (FEWS) can be considered as an advancement of the ATR technique, where the sensitivity is enhanced by the increased number of internal reflections.^8^ Since the intensity of the evanescent field strongly depends on the waveguide geometry, tapering a section of the fiber or bending it, further increases the number of internal reflections, and the penetration depth of the evanescent field into the measured sample, thus improving the sensitivity of detection.^9,10^

Mid-IR transmitting fibers are typically fabricated from a variety of materials, such as polycrystalline silver halides, amorphous chalcogenide glasses, fluoride glasses and tellurium halides. Among these, silver halides have many advantages for developing mid-IR FEWS systems due to their flexibility, transparency in the entire mid-IR spectral range, being non-toxic and insoluble in water and in many organic solvents.^11^

Several mid-IR evanescent-field based sensors with enhancement coatings for measuring compounds in aqueous solutions have been developed and discussed.^12–20^ Yet, the detection of VOCs in the gas-phase in air (or in other gases) through evanescent-field sensing still remains a great challenge. The reason is that gas is a low-density matrix (approximately three orders of magnitude lower in density than liquid or solid matrices), resulting in a small number of molecules at the fiber surface, at any given time, and consequently causing a low efficiency for these sensors operating in the gas phase. Therefore, evanescent-field based mid-IR detection of VOCs in gas-phase is uncommon and particularly challenging. Recently, Jin *et al.* demonstrated evanescent-field detection of ethanol at the C–H absorption band, using a single-mode chalcogenide waveguide.^21^ This work demonstrates the principle of selective evanescent-field detection in the gas-phase, however, the microfabrication process is complex and single-mode operation limits the spectral range of the measurement.

Functionalized coatings have been used to adsorb and concentrate VOCs from gas-phase to enable better detection for monitoring refractive index changes.^22–25^ Although sensitive measurement of refractive index changes was successfully achieved by some of these methods, they do not allow molecular specificity. Nano-porous materials, such as porous silicon (pSi), have been shown to be highly useful in adsorbing and condensing VOCs due to capillary action in the nanopores of the pSi.^26–28^

**Fig. 1:**
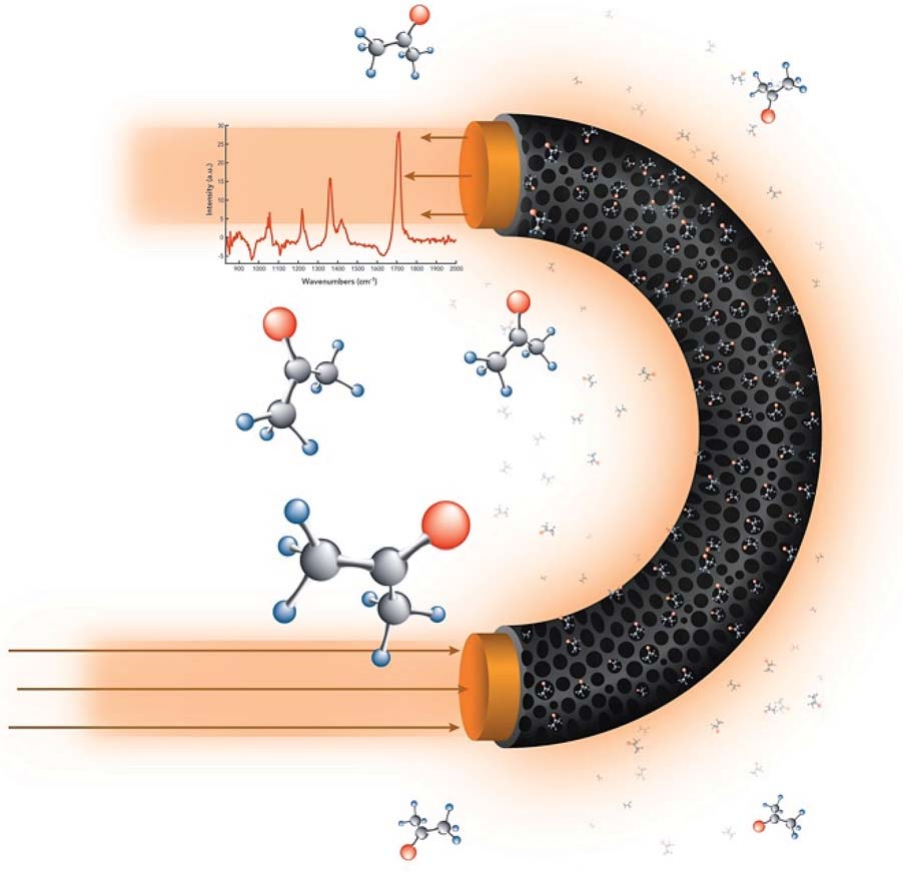
Schematic principle of an optical fiber sensor coated with a porous cladding, capable of concentrating volatile molecules near its surface in order to increase the number of molecules interacting with the evanescent field.

In this paper, we report a mid-IR based evanescent-field sensor which shows significant sensitivity enhancement towards VOCs in the gas-phase. The sensing principle is based on a nano-porous cladding which is capable of concentrating volatile molecules near the surface of the fiber sensor, thus increasing the number of molecules interacting with the evanescent field – Fig. 1. We have demonstrated this effect by coating a U-bent region of a silver-halide fiber with nano-porous silicon microparticles and performing spectroscopic analysis on different analytes. This sensing arrangement provides a platform for the development of practical sensors for VOC detection in the gas-phase.

## Experimental

### Preparation of a U-bent fiber

The measurements in this work were carried out using silver-halide (AgBrCl) fibers. The exact fabrication procedure, as well as the mechanical, physical and optical properties of these fibers, are discussed in detail elsewhere.^29,30^ Briefly, a single crystal is first grown from a hot melt of AgCl and AgBr at a predefined concentration of each constituent, and then is extruded through a die, to form polycrystalline fibers of a desired diameter. A U-bend with a radius of approximately 0.5 cm was prepared by bending the fiber gently around a small cylinder. The two ends of the fiber were coupled to a Fourier-transform infra-red (FTIR) spectrometer (ArcOptix, Switzerland).

### Fabrication of the pSi microparticles and coating of the fiber

Nano-porous silicon membranes with a diameter of 15 mm were fabricated from a boron-doped silicon wafer with a resistivity of 0.01–0.02 Ω cm and (100) crystal orientation. The silicon wafer was electrochemically etched in 3 : 7 mixture of 48% hydrofluoric acid and ethanol under a current density of 25 mA cm^−2^ for 50 minutes. In order to detach the porous layer, a current of 500 mA was applied for 30 seconds.

The pSi microparticles were made by breaking up a nano-porous silicon membrane into small particles *via* sonication in an 80% solution of ethanol in deionized (DI) water for 10 minutes. The resulting microparticle suspension was pipetted onto the U-bent region of the fiber and heated at a distance of ∼10 cm above a hot-plate held at 120 °C until all of the ethanol–DI water solution evaporated, leaving behind the pSi microparticles physisorbed onto the fiber-surface. During the heating process, a rotating motion was manually applied to the fiber to evenly distribute the pSi particles across its surface. This coating process, including the microparticle deposition and solvent evaporation, can be repeated more than once to ensure a full and uniform coverage of the U-bent region of the fiber. A field-emission scanning electron microscope (FEG-SEM, TESCAN MIRA3, X-Max, Oxford Instruments) was used to take images, which were processed using ImageJ software to obtain information on the coverage, dimensions and morphology of the pSi microparticles.

### Sensor setup and optical measurements

**Fig. 2:**
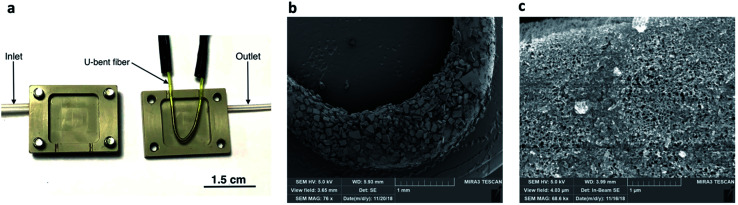
(a) Picture of a pSi microparticle-coated fiber in an open gas cell; (b) SEM image of pSi microparticles on the surface of a silver-halide fiber; (c) SEM image of a single pSi microparticle.

A custom-built gas flow-cell was designed for carrying out VOC sensing using the fiber sensor (Fig. 2a and S1 in the ESI† for further details). The 26 × 20 × 8 mm flow-cell comprised of two polyether ether ketone (PEEK) parts, each with a 15 × 15 × 2.5 mm internal cavity. The two compartments of the flow-cell were sealed together using four bolts with the U-bent fiber inside, ensuring that the sensitive portion of the fiber was suspended within the gas compartment an internal volume of approximately 1.1 cm^3^. Lateral holes on either side of the chamber were used as the inlet and outlet for VOCs, flowing in one direction. The flow of VOCs through the gas cell was obtained by bubbling pure air through bottled VOC liquids on a temperature-controlled plate (Torrey Pines Scientific, Echotherm). The resulting vapor was directed through the outlet of the sealed bottle into the inlet of the gas chamber (containing the U-bent fiber) through connecting tubing. The flow of air and VOCs were controlled using flow controllers (Alicat Scientific) and was set to 500 sccm. Fig. S1, in the ESI,† shows schematic diagrams of the experimental setups used in this study.

## Results and discussion

### Fiber coating and SEM analysis

The photo of the U-bent silver-halide sensing fiber placed in the open flow-cell is shown in Fig. 2a. Fig. 2b shows an SEM image of a pSi microparticle coating adhering to the U-bent region of the fiber surface, with microparticle dimensions ranging from 20 to 200 μm. These dimensions are likely to be highly dependent on the membrane sonication time. The microparticle coverage of the fiber was controlled by the number of coating cycles applied and rotation speed. Fig. 2c shows a zoomed-in SEM image of a single pSi microparticle, illustrating the nanopores, with dimensions ranging between 50 and 500 nm.

Ideally, the fiber coating should not significantly reduce the optical mid-IR transmission of the fiber, otherwise the optical losses will be high and sensor performance will be compromised.

### Optimization of the number of coatings

**Fig. 3:**
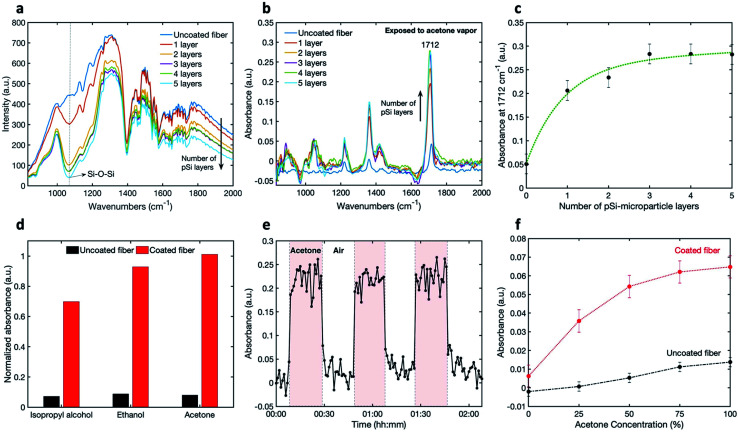
(a) Infrared transmission spectra of a U-bent silver-halide fiber with no coating and with one to five coating layers of pSi microparticles; (b) absorbance spectra of an uncoated fiber and of the same fiber coated with increasing layers of pSi microparticles, exposed to acetone vapor; (c) absorbance intensity of the C=O acetone stretch at 1712 cm^−1^ as a function of the number of pSi layers; (d) normalised absorbance peak intensity of isopropyl alcohol (945 cm^−1^), ethanol (1045 cm^−1^) and acetone (1712 cm^−1^) for uncoated and coated fibers; (e) time-dependent measurement at 1712 cm^−1^ when switching from air to saturated acetone vapor three times and (f) absorbance of the C=O acetone stretch at 1712 cm^−1^ as a function of acetone concentration for uncoated and coated fibers.

To determine the transmission and the light attenuation of mid-IR radiation in the coated U-bent fiber, we performed 5 iterations of coating the fiber and then measured the transmitted spectrum in air (Fig. 3a). These spectra show the effect of increased absorbance upon adding pSi layers on the fiber, as well as peaks associated with the silver-halide fiber (described elsewhere), atmospheric absorption, and absorption by the components of the FTIR spectrometer.^29^ The raw measured spectrum of the pSi-coated fiber shows a slight overall decrease in intensity compared to the uncoated fiber, and a prominent absorption band of a coated fiber at 1070 cm^−1^, corresponding to a Si–O–Si stretch, pointing to oxidized pSi microparticles.^31–34^ Indeed, microparticles oxidize far more rapidly than bulk Si due to a larger surface area. Compared to the uncoated fiber, the overall fiber transmission decreased by 5% to 38% with 1 to 5 coating-layers respectively. To see the effect of the number of coatings on the measured IR absorbance spectra, we exposed the uncoated and the coated fibers to saturated acetone vapor (Fig. 3b). It can be seen that there is an increase in the absorbance peak intensity for the coated fibers, compared to the uncoated fiber.

The peak intensity at 1712 cm^−1^, corresponding to the acetone carbonyl C=O stretch, is plotted as a function of the number of layers in Fig. 3c.

The absorbance is seen to increase with the number of pSi microparticle layers until saturation is reached after the third layer. This saturation may be caused by a reduction of analyte penetration close to the fiber through a thick set of pSi layers.

### Operational sensitivity

Once the optimal number of pSi coatings was established, the detection sensitivity of the coated fibers *versus* uncoated, was assessed. For this purpose, the vapors of ethanol and isopropyl alcohol were also measured, showing the expected mid-IR peaks (see Fig. S2 in the ESI†).

The enhancement in sensitivity of the coated fiber, as compared to an uncoated fiber, is shown in Fig. 3d for isopropyl alcohol, ethanol and acetone, where the absorbance-peak intensities are plotted. The selected peaks are at 945 cm^−1^ for isopropyl alcohol, 1045 cm^−1^ for ethanol and 1712 cm^−1^ for acetone. Based on these results, the factors of enhancement in sensitivity (Abs_coated_/Abs_uncoated_) for acetone, ethanol and isopropyl alcohol were determined to be 12.7, 10.5 and 9.6 respectively, demonstrating an order-of-magnitude of sensitivity enhancement using this method. The enhancement is not quite the same for the three measured compounds, probably because the VOCs adsorption onto the nano-porous matrix is highly dependent on the analyte properties, such as its affinity to the surface and the analyte vapor pressure.

In addition to the enhancement in the sensor sensitivity, other performance characteristics, such as kinetic response and recovery times, are very important. In order to demonstrate the reversibility of sensor's response, we conducted time-dependent measurements by repeatedly exposing the sensor to VOCs. Each spectrum was integrated over one minute. Fig. 3e shows the absorbance intensity at 1712 cm^−1^ for acetone, when switching from air to saturated acetone vapor three times. It can be seen, that the absorbance signal returns to the original baseline level throughout, meaning that the sensor shows good reversibility. Moreover, the response and recovery times appear to be around one minute.

Next, the absorbance signal was measured as a function of VOC concentration for uncoated and coated fibres using the setup shown in Fig. S1c in the ESI.†Fig. 3f shows the results of gradually increasing concentrations of acetone through the gas flow-cell from 0 to 100%, for both uncoated and coated fibers. An increase in the absorbance can be observed with an increase of acetone concentration for both fibers. This increase is significantly larger for the coated fiber. The plateau response of the coated fibers, visible at the high concentrations, could be due to pore saturation. The error bars were calculated by taking into account the changes in the intensity at a specific wavelength over time and calculating the respective standard deviation.

### Mixtures

**Fig. 4:**
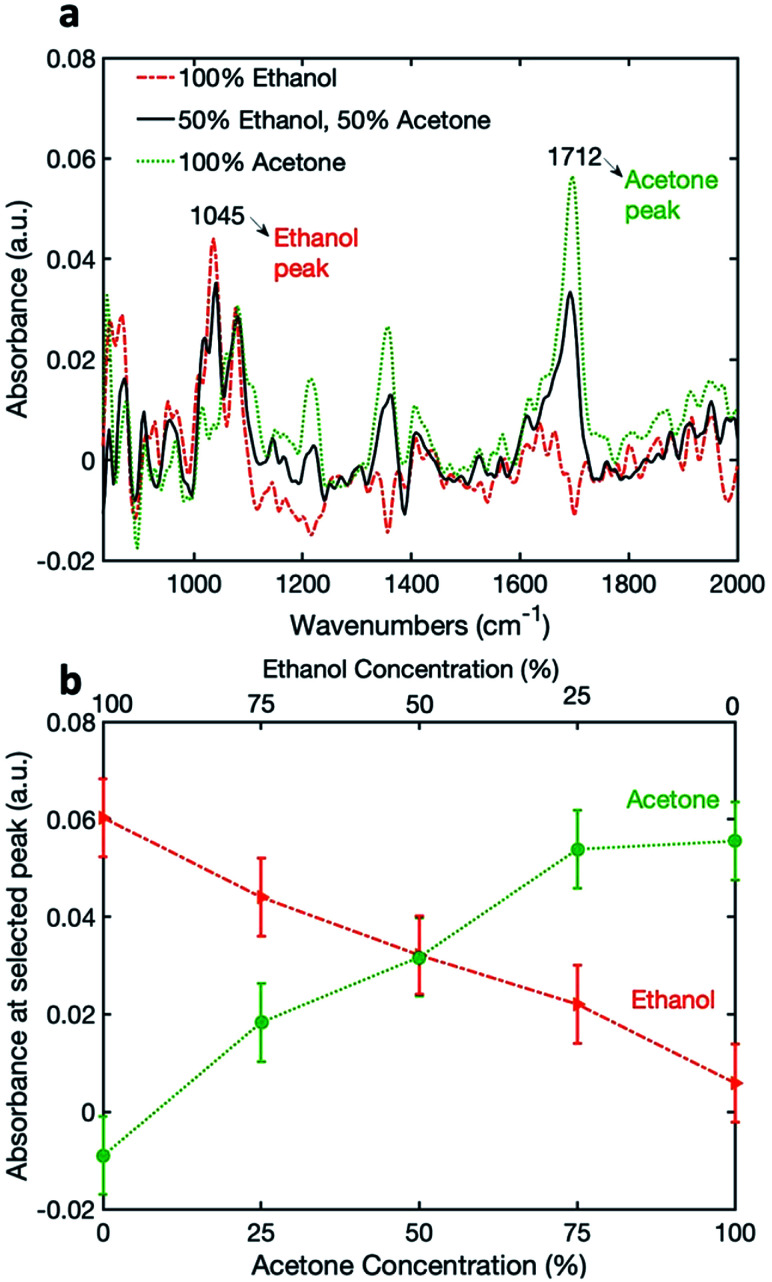
(a) Absorbance spectra of 100% acetone, 100% ethanol and a 50% : 50% mixture using a pSi microparticle coated fiber; (b) simultaneous measurement of ethanol and acetone using a pSi microparticle-coated fiber, showing the absorbance at wavelengths of 1045 cm^−1^ and 1712 cm^−1^ for ethanol and acetone, respectively, at different concentrations.

Finally, in order to demonstrate the capability for simultaneous detection, the coated pSi fiber was exposed to a mixture of ethanol and acetone. The relative concentrations of ethanol and acetone were simultaneously varied between 0 and 100% (see Table S1 in the ESI†). The absorbance spectra of 100% acetone, 100% ethanol and a 50% : 50% mixtures are shown in Fig. 4a, with the peaks chosen for analysis being 1045 cm^−1^ and 1712 cm^−1^ for ethanol and acetone, respectively. Fig. 4b shows the variation of absorbance at the selected wavelengths for acetone and ethanol, as a function of relative vapor concentrations. It can be seen that the signal for acetone increases with the increasing relative acetone concentration, and the signal for ethanol decreases with the decrease in relative ethanol concentration. For acetone, we see a similar plateau response as previously, while for ethanol the response seems to be linear over the measured range. This can be attributed to the difference between the saturated vapor pressures of acetone and ethanol at 20 °C: 184 mmHg *vs.* 43.7 mmHg, respectively.^35^ Therefore, we may conclude that a high vapor concentration of acetone saturates the pSi nano-pores.

## Conclusions

A novel fiber-optic evanescent wave spectroscopy method was found to enhance the sensitivity for detecting VOCs. In this design, the surface of a waveguide or an optical fiber is coated with a nano-porous cladding which concentrates VOCs inside it, thus enabling a larger number of molecules to interact with the evanescent field of the fiber sensor.

This concept has been demonstrated using a silver-halide U-bent fiber, coated with nano-porous silicon microparticles. The effect of the microparticles on the optical IR transmission spectrum was studied. We showed that the pSi-coated U-bent fiber exhibited a significantly higher sensitivity for the three measured VOCs – isopropyl alcohol, ethanol and acetone. We also demonstrated the simultaneous measurement of different relative concentrations of acetone and ethanol.

This arrangement can be further improved in enabling better sensitivity and selectivity by functionalizing the surface of the pSi and hence changing the affinity of the different VOCs to the nano-porous surface. We believe that the sensitivity could be further optimized by varying the dimensions and density of microparticles and the dimensions of the nano-pores, as well as by improving the homogeneity of the coating and by a more controlled variation of the size of the coated region of the fiber. Our future work will focus on exploring the aforementioned possibilities.

## Conflicts of interest

There are no conflicts of interest to declare.

## Supplementary Material

RA-009-C9RA04104D-s001
