# Supplementary material for: Mid-IR evanescent-field fiber sensor with enhanced sensitivity for volatile organic compounds
Source: RSC Adv. 2019 Jul 8;9(37):21186–91. doi: 10.1039/c9ra04104d (PMC9066189; doi:10.1039/c9ra04104d)
Supplement: RA-009-C9RA04104D-s001 [file RA-009-C9RA04104D-s001.pdf]

Electronic Supplementary Material (ESI) for RSC Advances.
This journal is © The Royal Society of Chemistry 2019

Mid-IR Evanescent-field Fiber Sensor with Enhanced Sensitivity for Volatile Organic Compounds

Farah Alimagham, Max Platkov, Joshua Prestage, Svetlana Basov, Gregory Izakson, Abraham Katzir, Stephen R. Elliott, and Tanya Hutter

Supplementary material

S1. Schematic diagrams of the experimental setups for carrying out VOC sensing

Figure S1 shows schematic diagrams of the experimental setups for carrying out VOC sensing using the fiber sensor. The setup in Figure S1b was used for comparing the sensitivity of an uncoated U-bent fiber with that of the same fiber coated with pSi, as well as for determining the number of pSi coatings for optimal sensitivity. For these experiments, a 4-point valve was used to switch between pure air, air containing VOCs at their saturated vapor pressure (isopropanol alcohol, acetone and ethanol) at different concentrations flowing through the cell.

The setup in Figure S1c was used for testing the qualitative sensing capabilities of the sensor by conducting simultaneous measurements of two different VOCs at varying concentrations. In this case, the relative concentration of each VOC was controlled by their flow-rate through the gas chamber at a constant (room) temperature. Here, a T-junction valve was used to mix the two different VOCs at different flow-rates, varying from 0 to 500 sccm (standard cubic cm per minute), resulting in different VOC mixtures (see Table S1). Initially, a mixture of acetone and air were measured using an uncoated and coated fiber, followed by the simultaneous measurement of acetone and ethanol at varying concentrations.

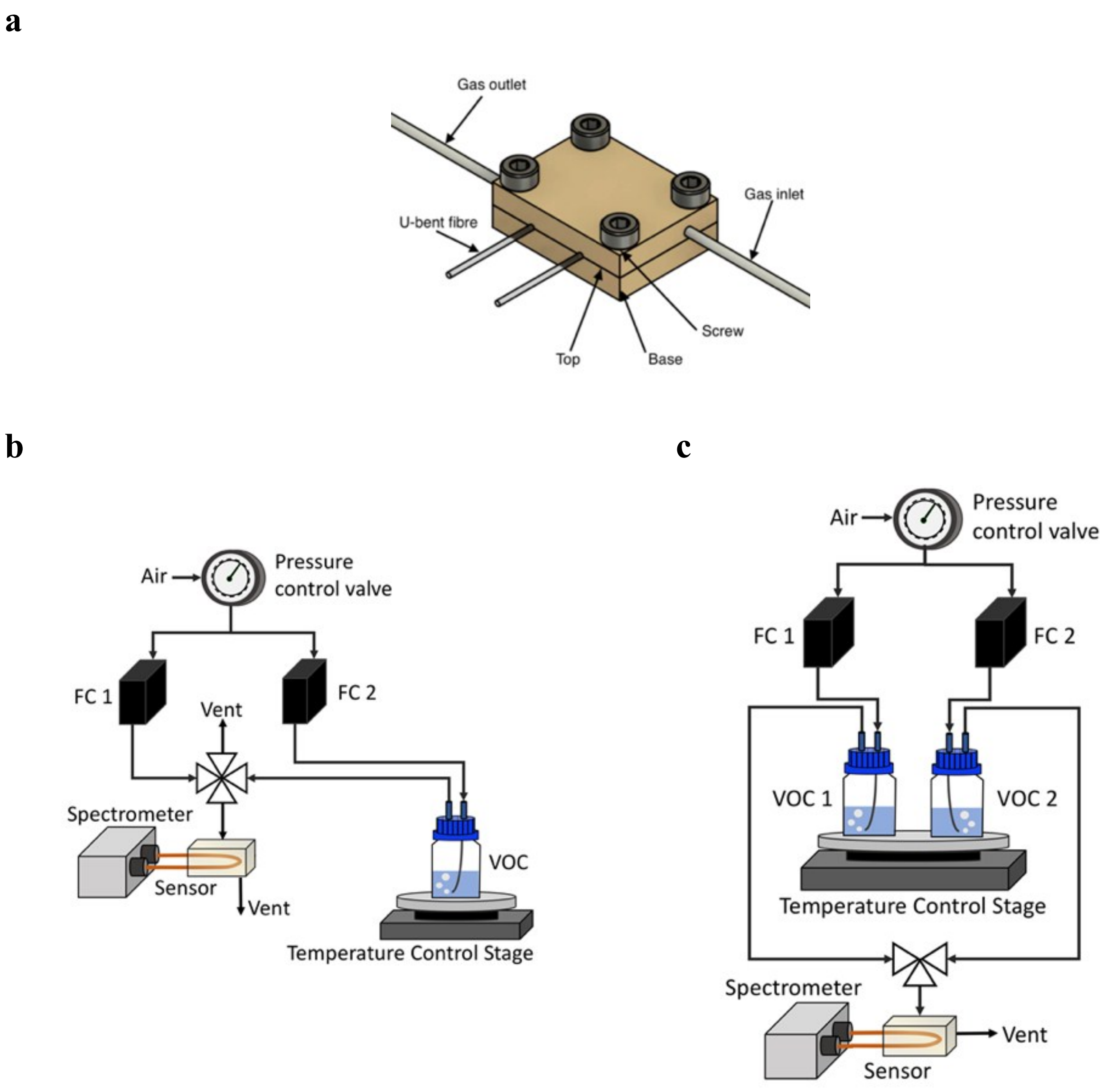

Figure S1. Schematic illustration of the experimental setup for VOC sensing. (a) VOC sensor comprising of a gas flow cell with an internal cavity for the U-bent fiber; (b) Setup for detection of pure VOCs at different concentrations; (c) Setup for simultaneous detection of two VOCs at varying concentrations. The gas flow rate is controlled by two flow controllers FC1 and FC2.

S2. Mid-IR measurements of ethanol and isopropyl alcohol vapours on bare and coated fibers

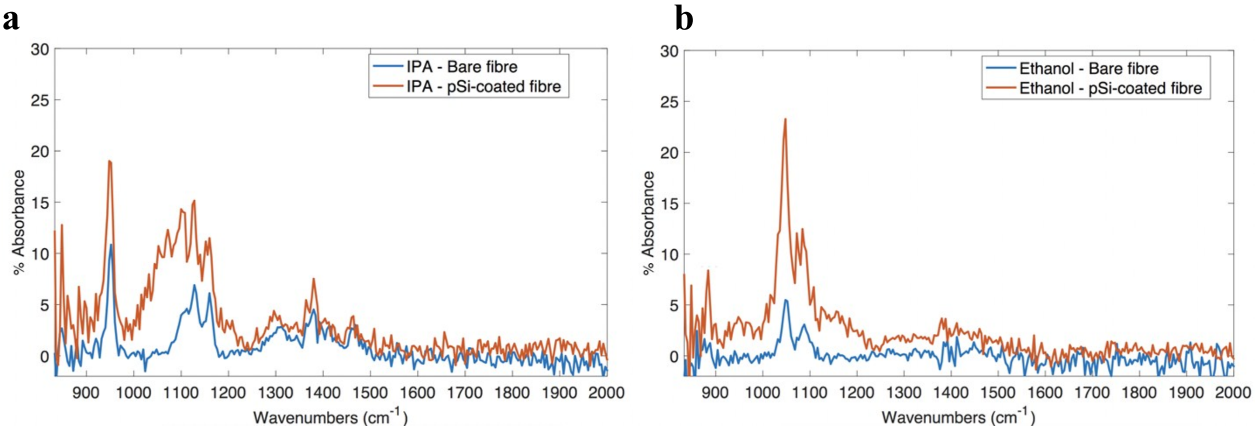

Figure S2. Spectra of coated and uncoated fibers when exposed to (a) isopropyl alcohol and (b) ethanol.

S3. Relative concentrations of ethanol and acetone used in this study

Table S1. Flow-controller flow-rates to achieve different mixtures of VOC_1 and VOC_2 flowing through gas chamber.

FC1 flow-rate (sccm)	VOC_1 (%)	FC2 flow-rate (sccm)	VOC_2 (%)
500	100	0	0
400	75	100	25
250	50	250	50
100	25	400	75
0	0	500	100
